# Supplementary material for: Patterns of Intron Gain and Loss in Fungi
Source: PLoS Biol. 2004 Nov 30;2(12):e422. doi: 10.1371/journal.pbio.0020422 (PMC532390; doi:10.1371/journal.pbio.0020422)
Supplement: Table S1 — Also available at http://genes.mit.edu/NielsenEtAl/. (4.3 MB ZIP). [file pbio.0020422.st001.zip › NielsenEtAl/html/978.html]

AN4803.1.NCU01949.1.MG02952.1.FG08896.1


```
 CLUSTAL W (1.82) Multiple Sequence Alignments - Introns Inserted


Sequence 1: NCU01949.1	190 aa
Sequence 2: MG02952.1	191 aa
Sequence 3: FG08896.1	191 aa
Sequence 4: AN4803.1	193 aa
Alignment Length: 193 aa
Number Identitical Residues: 159 aa
Alignment Score (without introns) 6261


MG02952.1 	--MVR~PSYSKTAK2VPRRP~FEAARL2DSELKLVGEFGLKNKREVWRVQLTLSKIRRAA
NCU01949.1	--MAP~RSYSKTAK~VPRRP~FEAARL2DSELKLVGEYGLRNKREVWRVLLTLSKIRRAA
FG08896.1 	--MPP~RAHSKTSR~VPRRP~FESARL2DSELKLVGEYGLRNKREVWRVGLTLSKIRRAA
AN4803.1  	MAPVP2RVYSKTYK~VPRRP1FESARL2DSELKIVGEYGLRNKREVWRVQFTLSKIRRAA
          	 :      :*** : ***** **:*** *****:***:**:******** :*********

MG02952.1 	R2QLLTLDEKDPKRLFEGNALIRRLVRVGVLDESRMKLDYVLALKVEDFLERRLQTCVYK
NCU01949.1	R2QLLTLDEKDPKRLFEGNALIRRLVRVGVLDESRMKLDYVLALKAEDFLERRLQTCVYK
FG08896.1 	R2QLLTLDEKDPKRLFEGNALIRRLVRVGVLDESRMKLDYVLALKIEDFLERRLQTCVWK
AN4803.1  	R2ELLTLDEKDPKRLFEGNALIRRLVRIGVLDESRMKLDYVLALRVEDFLERRLQTCVYK
          	* :************************:****************: ************:*

MG02952.1 	LGLAKSIHHARVLIRQRHIRVGKQIVNVPSFVVRLDSQKHIDFALTSPFGGGRPGRVRRK
NCU01949.1	LGLAKSIHHARVLIRQRHIRVGKQIVNVPSFVVRLDSQKHIDFALTSPFGGGRPGRVRRK
FG08896.1 	LGLAKSIHHARVLIRQRHIRVGKQIVNVPSFIVRLDSQKHIDFALTSPFGGGRPGRVRRK
AN4803.1  	LGLAKSIHHARVLIKQRHIRVGKQIVNVPSYMVRLDSQKHIDFALTSPFGGGRPGRVQRK
          	**************:***************::*************************:**

MG02952.1 	KAKSAEGGEDAGDDEDEE
NCU01949.1	KAKAAEGGE-AAEEEDEE
FG08896.1 	KAKAAEGGEGGEDEEDEE
AN4803.1  	KAAAAAGGGDDEGEEDEE
          	** :* ** .   :****
```
